# 4,4′-(*o*-Phenyl­enedioxy­dimethyl­ene)dipyridinium dinitrate

**DOI:** 10.1107/S1600536809040215

**Published:** 2009-10-10

**Authors:** Ping Zou, Shuang Zhang, Ying Liu, Jun Qiao, Jin-Sheng Gao

**Affiliations:** a College of Life Science, Sichuan Agricultural University, Ya’an 625014, People’s Republic of China; b College of Chemistry and Materials Science, Heilongjiang University, Harbin 150080, People’s Republic of China

## Abstract

The cation of the salt, C_18_H_18_N_2_O_2_^2+^·2NO_3_^−^, lies about a twofold rotation axis. The pyridinium ring is almost coplanar with the phenyl­ene ring [dihedral angle between rings = 5.69 (9)°]. The crystal structure shows π–π stacking inter­actions [centroid–centroid distance = 3.70 (1) Å] between the pyridinium rings and the phenyl­ene rings, generating a linear chain structure. The cation also forms two N—H⋯O hydrogen bonds to two nitrate groups.

## Related literature

For general background to the title compound, see: Siaw-Lattey *et al.* (2005 ▶); Burchell *et al.* (2006 ▶). For the synthesis, see: Gao *et al.* (2004 ▶). For related structures, see Gao *et al.* (2006 ▶, 2009*a* ▶,*b* ▶).
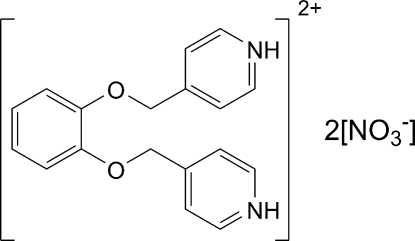


## Experimental

### 

#### Crystal data

C_18_H_18_N_2_O_2_^2+^·2NO_3_^−^
*M* *_r_* = 418.36
Monoclinic, 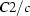

*a* = 10.364 (6) Å
*b* = 19.7593 (11) Å
*c* = 9.996 (8) Å
β = 110.75 (2)°
*V* = 1914.2 (19) Å^3^
*Z* = 4
Mo *K*α radiation
μ = 0.12 mm^−1^
*T* = 291 K
0.40 × 0.22 × 0.16 mm

#### Data collection

Rigaku R-AXIS RAPID diffractometer
Absorption correction: multi-scan (*ABSCOR*; Higashi, 1995 ▶) *T*_min_ = 0.955, *T*_max_ = 0.981
9339 measured reflections
2195 independent reflections
1203 reflections with *I* > 2σ(*I*)
*R*_int_ = 0.049

#### Refinement

*R*[*F* ^2^ > 2σ(*F* ^2^)] = 0.055
*wR*(*F* ^2^) = 0.144
*S* = 1.03
2195 reflections
140 parameters
18 restraints
H atoms treated by a mixture of independent and constrained refinement
Δρ_max_ = 0.28 e Å^−3^
Δρ_min_ = −0.17 e Å^−3^

### 

Data collection: *RAPID-AUTO* (Rigaku, 1998 ▶); cell refinement: *RAPID-AUTO*; data reduction: *CrystalClear* (Rigaku/MSC, 2002 ▶); program(s) used to solve structure: *SHELXS97* (Sheldrick, 2008 ▶); program(s) used to refine structure: *SHELXL97* (Sheldrick, 2008 ▶); molecular graphics: *SHELXTL* (Sheldrick, 2008 ▶); software used to prepare material for publication: *SHELXL97*.

## Supplementary Material

Crystal structure: contains datablocks I, global. DOI: 10.1107/S1600536809040215/ng2647sup1.cif

Structure factors: contains datablocks I. DOI: 10.1107/S1600536809040215/ng2647Isup2.hkl

Additional supplementary materials:  crystallographic information; 3D view; checkCIF report

## Figures and Tables

**Table 1:** Hydrogen-bond geometry (Å, °)

*D*—H⋯*A*	*D*—H	H⋯*A*	*D*⋯*A*	*D*—H⋯*A*
N1—H10⋯O2	0.92 (3)	2.34 (3)	3.017 (3)	130 (2)
N1—H10⋯O3	0.92 (3)	1.96 (3)	2.873 (3)	170 (3)

**Fig. 1.**
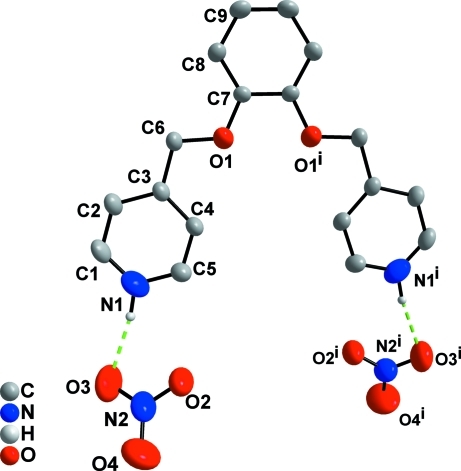
The molecular structure of the title compound, showing displacement ellipsoids at the 50% probability level for non-H atoms. [Symmetry codes: (i) 2 - x, y, 2.5 - z]

**Fig. 2.**
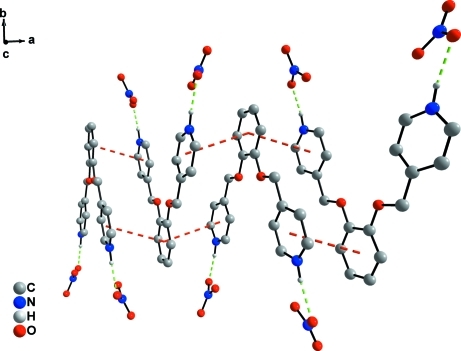
A partial packing view, showing the one-dimensional chain. Green dashed lines indicate the hydrogen bonds and red dashed lines indicate the πi—πi stacking interactions.

## supplementary crystallographic information

### Comment

Many poly-N-heterocyclic ligands coordinated with transition metal ions can form a variety of topology structures, including macrocycles, polyhedra and linear and helical polymers. Son's group have reported the synthesis of bis(pyridylether) ligand, which reacted with AgNO_3_, Cu(ClO_4_)_2_ and Co(NCS)_2_ to produce a helical metallopolymer, a bridged dinuclear complex and a monomeric octahedral complex, respectively. Puddephatt's group have investigated a series of silver complexes of two U-shaped bis(amidopyridyl) ligands, which assemble into macrocyclic and one-dimensional chain that are connected further into two- or three-dimensional structures by anion binding and hydrogen bonding. Our group has report three kinds of flexible pyridyl-based ligands in the previous report (Gao *et al.* 2006; Gao *et al.* 2009*a*; Gao *et al.* 2009*b*). As a part of our continuing research for bipyridyl aromatic ligands, we report the crystal structure of the title compound here.

In the title compound, the diprotonated 1,2-bis(4-pyridylmethoxy)benzene cation is centrosymmetric. The two terminal pyridyl rings lie in an almost coplane arrangement with the central benzene ring [dihedral angles of 5.69 (9)°]. The dihedral angle between the two pyridyl rings is 10.22 (8)° (Figure 1).

In the crystal packing structure, the πi—πi stacking interactions [distance of 3.70 (1) Å] existing between each spacer benzene ring and two adjacent pyridine rings from different ligands link the ligands into a one-dimensional chain structure. Furthermore, the uncoordinated nitrate anions are stabilized by the C—H···O hydrogen bonds (Figure 2, Table 1).

### Experimental

The 1,2-bis(4-pyridylmethoxy)benzene was synthesized by the reaction of *o*-benzenediol and 4-chloromethylpyridine hydrochloride under nitrogen atmosphere and alkaline condition (Gao *et al.*, 2004; Gao *et al.*, 2006). Colorless block-shaped crystals of the title compound were obtained by slow evaporation of an ethanol solution after several days.

### Refinement

H atoms bound to C atoms were placed in calculated positions and treated as riding on their parent atoms, with C—H = 0.93 Å (aromatic), C—H = 0.97 Å (methylene), and with *U*_iso_(H) = 1.2*U*_eq_(C). N-bond H atoms were located in a difference Fourier map and were refined freely.

### Crystal data

**Table:**

C_18_H_18_N_2_O_2_^2+^·2NO_3_^−^	*F*(000) = 872
*M**_r_* = 418.36	*D*_x_ = 1.452 Mg m^−^^3^
Monoclinic, *C*2/*c*	Mo *K*α radiation, λ = 0.71073 Å
Hall symbol: -C 2yc	Cell parameters from 5255 reflections
*a* = 10.364 (6) Å	θ = 3.0–24.5°
*b* = 19.7593 (11) Å	µ = 0.12 mm^−^^1^
*c* = 9.996 (8) Å	*T* = 291 K
β = 110.75 (2)°	Block, brown
*V* = 1914.2 (19) Å^3^	0.40 × 0.22 × 0.16 mm
*Z* = 4	

### Data collection

**Table:**

Rigaku R-AXIS RAPID diffractometer	2195 independent reflections
Radiation source: fine-focus sealed tube	1203 reflections with *I* > 2σ(*I*)
graphite	*R*_int_ = 0.049
ω scans	θ_max_ = 27.5°, θ_min_ = 3.0°
Absorption correction: multi-scan (*ABSCOR*; Higashi, 1995)	*h* = −13→13
*T*_min_ = 0.955, *T*_max_ = 0.981	*k* = −23→25
9339 measured reflections	*l* = −12→12

### Refinement

**Table:**

Refinement on *F*^2^	Primary atom site location: structure-invariant direct methods
Least-squares matrix: full	Secondary atom site location: difference Fourier map
*R*[*F*^2^ > 2σ(*F*^2^)] = 0.055	Hydrogen site location: inferred from neighbouring sites
*wR*(*F*^2^) = 0.144	H atoms treated by a mixture of independent and constrained refinement
*S* = 1.03	*w* = 1/[σ^2^(*F*_o_^2^) + (0.0652*P*)^2^ + 0.3119*P*] where *P* = (*F*_o_^2^ + 2*F*_c_^2^)/3
2195 reflections	(Δ/σ)_max_ < 0.001
140 parameters	Δρ_max_ = 0.28 e Å^−^^3^
18 restraints	Δρ_min_ = −0.17 e Å^−^^3^

### Special details

**Table:**

Geometry. All e.s.d.'s (except the e.s.d. in the dihedral angle between two l.s. planes) are estimated using the full covariance matrix. The cell e.s.d.'s are taken into account individually in the estimation of e.s.d.'s in distances, angles and torsion angles; correlations between e.s.d.'s in cell parameters are only used when they are defined by crystal symmetry. An approximate (isotropic) treatment of cell e.s.d.'s is used for estimating e.s.d.'s involving l.s. planes.
Refinement. Refinement of *F*^2^ against ALL reflections. The weighted *R*-factor *wR* and goodness of fit *S* are based on *F*^2^, conventional *R*-factors *R* are based on *F*, with *F* set to zero for negative *F*^2^. The threshold expression of *F*^2^ > σ(*F*^2^) is used only for calculating *R*-factors(gt) *etc*. and is not relevant to the choice of reflections for refinement. *R*-factors based on *F*^2^ are statistically about twice as large as those based on *F*, and *R*- factors based on ALL data will be even larger.

### Fractional atomic coordinates and isotropic or equivalent isotropic displacement parameters (Å^2^)

**Table:**

	*x*	*y*	*z*	*U*_iso_*/*U*_eq_	
C1	0.8168 (3)	0.12902 (14)	0.7346 (3)	0.0648 (7)	
H1	0.7836	0.1178	0.6380	0.078*	
C2	0.8406 (2)	0.19528 (14)	0.7751 (2)	0.0601 (7)	
H2	0.8229	0.2291	0.7063	0.072*	
C3	0.8913 (2)	0.21178 (12)	0.9188 (2)	0.0493 (6)	
C4	0.9127 (3)	0.16022 (12)	1.0166 (3)	0.0600 (7)	
H4	0.9450	0.1699	1.1140	0.072*	
C5	0.8867 (3)	0.09496 (13)	0.9712 (3)	0.0668 (7)	
H5	0.9012	0.0602	1.0377	0.080*	
C6	0.9194 (3)	0.28409 (12)	0.9634 (2)	0.0553 (6)	
H6A	0.9925	0.3017	0.9339	0.066*	
H6B	0.8372	0.3111	0.9188	0.066*	
C7	0.9788 (2)	0.34991 (10)	1.1756 (2)	0.0460 (5)	
C8	0.9581 (2)	0.41020 (12)	1.1024 (3)	0.0546 (6)	
H8	0.9299	0.4104	1.0031	0.066*	
C9	0.9797 (3)	0.47065 (11)	1.1773 (3)	0.0580 (6)	
H9	0.9663	0.5115	1.1281	0.070*	
N1	0.8408 (2)	0.08066 (13)	0.8325 (2)	0.0632 (6)	
H10	0.830 (3)	0.0347 (17)	0.815 (3)	0.095 (10)*	
N2	0.7766 (2)	−0.08567 (12)	0.8502 (2)	0.0687 (6)	
O1	0.95913 (17)	0.28718 (7)	1.11369 (15)	0.0572 (5)	
O2	0.8126 (2)	−0.05377 (9)	0.96265 (19)	0.0749 (6)	
O3	0.7935 (2)	−0.05844 (11)	0.7449 (2)	0.0908 (7)	
O4	0.7253 (3)	−0.14159 (13)	0.8438 (3)	0.1233 (10)	

### Atomic displacement parameters (Å^2^)

**Table:**

	*U*^11^	*U*^22^	*U*^33^	*U*^12^	*U*^13^	*U*^23^
C1	0.0624 (16)	0.0818 (19)	0.0516 (15)	−0.0097 (14)	0.0218 (13)	−0.0211 (14)
C2	0.0659 (16)	0.0697 (16)	0.0447 (13)	−0.0076 (13)	0.0198 (12)	−0.0086 (12)
C3	0.0474 (12)	0.0563 (14)	0.0430 (12)	0.0003 (11)	0.0144 (10)	−0.0063 (10)
C4	0.0729 (16)	0.0537 (15)	0.0459 (13)	0.0010 (12)	0.0118 (12)	−0.0072 (11)
C5	0.0736 (18)	0.0551 (15)	0.0630 (16)	0.0005 (13)	0.0133 (13)	−0.0039 (13)
C6	0.0699 (15)	0.0541 (14)	0.0402 (12)	0.0008 (12)	0.0175 (11)	−0.0006 (10)
C7	0.0501 (12)	0.0382 (11)	0.0470 (11)	0.0006 (10)	0.0139 (10)	−0.0016 (10)
C8	0.0631 (15)	0.0488 (13)	0.0492 (13)	−0.0002 (11)	0.0164 (11)	0.0049 (11)
C9	0.0645 (15)	0.0406 (12)	0.0691 (15)	0.0020 (12)	0.0237 (13)	0.0076 (11)
N1	0.0598 (13)	0.0607 (15)	0.0666 (15)	−0.0034 (11)	0.0191 (11)	−0.0199 (12)
N2	0.0808 (16)	0.0634 (15)	0.0523 (14)	0.0062 (12)	0.0120 (12)	−0.0048 (12)
O1	0.0859 (11)	0.0411 (9)	0.0372 (8)	−0.0022 (8)	0.0125 (7)	−0.0021 (6)
O2	0.1068 (15)	0.0618 (11)	0.0505 (11)	−0.0016 (10)	0.0208 (10)	−0.0025 (9)
O3	0.1169 (16)	0.1030 (16)	0.0543 (12)	0.0114 (13)	0.0325 (11)	−0.0007 (11)
O4	0.160 (2)	0.0758 (15)	0.1154 (19)	−0.0354 (15)	0.0251 (16)	−0.0257 (13)

### Geometric parameters (Å, °)

**Table:**

C1—N1	1.326 (4)	C6—H6B	0.9700
C1—C2	1.367 (4)	C7—O1	1.368 (2)
C1—H1	0.9300	C7—C8	1.375 (3)
C2—C3	1.383 (3)	C7—C7^i^	1.394 (4)
C2—H2	0.9300	C8—C9	1.386 (3)
C3—C4	1.375 (3)	C8—H8	0.9300
C3—C6	1.494 (3)	C9—C9^i^	1.362 (5)
C4—C5	1.362 (3)	C9—H9	0.9300
C4—H4	0.9300	N1—H10	0.92 (3)
C5—N1	1.327 (3)	N2—O4	1.218 (3)
C5—H5	0.9300	N2—O2	1.226 (3)
C6—O1	1.411 (3)	N2—O3	1.249 (3)
C6—H6A	0.9700		
			
N1—C1—C2	120.3 (2)	C3—C6—H6B	110.1
N1—C1—H1	119.8	H6A—C6—H6B	108.4
C2—C1—H1	119.8	O1—C7—C8	125.0 (2)
C1—C2—C3	119.7 (2)	O1—C7—C7^i^	115.02 (11)
C1—C2—H2	120.1	C8—C7—C7^i^	119.93 (14)
C3—C2—H2	120.1	C7—C8—C9	119.6 (2)
C4—C3—C2	118.1 (2)	C7—C8—H8	120.2
C4—C3—C6	122.1 (2)	C9—C8—H8	120.2
C2—C3—C6	119.8 (2)	C9^i^—C9—C8	120.45 (14)
C5—C4—C3	120.1 (2)	C9^i^—C9—H9	119.8
C5—C4—H4	120.0	C8—C9—H9	119.8
C3—C4—H4	120.0	C1—N1—C5	121.4 (2)
N1—C5—C4	120.4 (3)	C1—N1—H10	126 (2)
N1—C5—H5	119.8	C5—N1—H10	112 (2)
C4—C5—H5	119.8	O4—N2—O2	120.0 (3)
O1—C6—C3	108.15 (19)	O4—N2—O3	122.4 (3)
O1—C6—H6A	110.1	O2—N2—O3	117.6 (2)
C3—C6—H6A	110.1	C7—O1—C6	117.47 (17)
O1—C6—H6B	110.1		

Symmetry codes: (i) −*x*+2, *y*, −*z*+5/2.

### Hydrogen-bond geometry (Å, °)

**Table:**

*D*—H···*A*	*D*—H	H···*A*	*D*···*A*	*D*—H···*A*
N1—H10···O2	0.92 (3)	2.34 (3)	3.017 (3)	130 (2)
N1—H10···O3	0.92 (3)	1.96 (3)	2.873 (3)	170 (3)
